# Characteristics and longitudinal progression of chronic obstructive pulmonary disease in GOLD B patients

**DOI:** 10.1186/s12890-017-0384-8

**Published:** 2017-02-20

**Authors:** Philip J. Lawrence, Umme Kolsum, Vandana Gupta, Gavin Donaldson, Richa Singh, Bethan Barker, Leena George, Adam Webb, Anthony J. Brookes, Christopher Brightling, Jadwiga Wedzicha, Dave Singh

**Affiliations:** 1 Centre for Respiratory Medicine and Allergy, Institute of Inflammation and Repair, Medicines Evaluation Unit, University Hospital of South Manchester Foundation Trust, University of Manchester, Manchester, UK; 2 Airways Disease Section, National Heart and Lung Institute, Imperial College London, London, UK; 3 Department of Infection, Immunity and Inflammation, Institute for Lung Health, NIHR Respiratory Biomedical Research, Leicester, UK; 4 Department of Genetics, University of Leicester, Leicester, UK

**Keywords:** Classification, Disease Progression, Symptoms

## Abstract

**Background:**

The characteristics and natural history of GOLD B COPD patients are not well described. The clinical characteristics and natural history of GOLD B patients over 1 year in a multicentre cohort of COPD patients in the COPDMAP study were assessed. We aimed to identify the subgroup of patients who progressed to GOLD D (unstable GOLD B patients) and identify characteristics associated with progression.

**Methods:**

Three hundred seventy COPD patients were assessed at baseline and 12 months thereafter. Demographics, lung function, health status, 6 min walk tests and levels of systemic inflammation were assessed. Students t tests and Mann Whitney-U tests were used.

**Results:**

One hundred seven (28.9%) of patients were categorised as GOLD B at baseline. These GOLD B patients had similar FEV1 to GOLD A patients (66% predicted). More GOLD B patients were current smokers (*p* = 0.031), had chronic bronchitis (*p* = 0.0003) and cardiovascular comorbidities (*p* = 0.019) compared to GOLD A. At 12 months, 25.3% of GOLD B patients progressed to GOLD D. These patients who progressed (unstable patients) had worse health status and symptoms (SGRQ-C Total, 50.0 v 41.1, *p* = 0.019 and CAT, 21.0 v 14.0, *p* = 0.006) and lower FEV_1_ (60% v 69% *p* = 0.014) at baseline compared to stable patients who remained in GOLD B.

**Conclusions:**

Unstable GOLD B patients who progressed to GOLD D had a higher level of symptoms at baseline. A high symptom burden may predict an increased likelihood of disease progression in GOLD B patients.

**Electronic supplementary material:**

The online version of this article (doi:10.1186/s12890-017-0384-8) contains supplementary material, which is available to authorized users.

## Background

Chronic obstructive pulmonary disease (COPD) is a heterogeneous condition, comprising different clinical and pathophysiological features that vary in both presence and severity between patients [1]. The Global Initiative for Chronic Obstructive Pulmonary Disease (GOLD) recommends a combined assessment to stratify patients into one of four categories (A/B/C/D) based on the severity of airflow limitation, degree of symptoms and exacerbation risk [1]. Patients with more symptoms are allocated into groups B or D and patients with high exacerbation risk and/or Forced Expiratory Volume in 1 s (FEV_1_) < 50% predicted are allocated into groups C or D. There are different pharmacological treatment recommendations for each category.

GOLD B patients are defined by mild to moderate airflow obstruction (FEV_1_ > 50%), a low exacerbation rate (<2 exacerbations per year and no hospitalisations) and a high burden of symptoms. Group B patients may deteriorate and change GOLD category; in the ECLIPSE cohort study, only 36% of group B patients remained stable after 1 year, 7% and 35% deteriorated to the higher risk categories C and D respectively, and 22% improved to group A [2]. The progression of group B patients may be due to FEV_1_ decline or an increase in exacerbations, or both.

COPD is often referred to as a progressive disease [1, 3]. However, the ECLIPSE study demonstrated that FEV_1_ did not change in some COPD patients over a 3 year follow up period [2]. The risk factors for greater FEV_1_ decline included current smoking, emphysema and exacerbations [4]. Recently, Lange, et al. [5], reported that the rate of FEV_1_ decline in COPD patients who have a low FEV_1_ in early adult life, indicating sub-optimal lung growth, is lower compared to COPD patients with normal FEV_1_ in early adult life (27 mls versus 52 mls/year respectively). These findings underscore the variation in lung function decline in COPD, influenced by multiple factors.

There appears to be heterogeneity within group B, as a subgroup of patients move over time into higher risk categories. We report a detailed characterisation of the natural history of GOLD B patients over 1 year in the COPDMAP cohort study. We describe the clinical features of group B compared to group A patients at the start of the study. We studied the stability of group B patients over 1 year, and describe the clinical characteristics of GOLD B patients who progressed to higher risk GOLD categories.

## Methods

### Study design

COPD patients aged 40 or over were recruited at 3 sites (Manchester, Leicester and Imperial/UCL) into the COPDMAP prospective observational cohort study (http://www.copdmap.org). All patients had a physician diagnosis of COPD, post-bronchodilator (post BD) FEV_1_/forced vital capacity (FVC) ratio <0.7 and ≥10 pack year smoking history. All patients provided written informed consent using protocols approved by the local Ethics Committees at each site (11/L0/1630; 10/H/1003/108; 07/H0406/157).

Stable visits were performed at baseline and at 6 month intervals up to 2 years. At the baseline visit, the demographic details and exacerbation history were collected. Symptoms and functional capacity were assessed and pulmonary function tests performed. Cardiovascular comorbidities were categorised as the following; body mass index (BMI) ≥25, stroke, peripheral vascular disease, high cholesterol, high blood pressure, heart attack, diabetes, atrial fibrillation, angina and other relevant cardiac events. Sputum and blood samples were obtained from patients at least 6 weeks after an exacerbation episode. These procedures were repeated at 6 monthly follow up visits; the yearly visit data is presented here. Exacerbation history throughout the observation period was based on patient recall to match how patients were classification at baseline. Patient recall agrees well with events detected with daily diary cards [6].

Patients were categorised as GOLD A, B, C or D using the 2016 GOLD guidelines [1]. Post bronchodilator FEV_1_ and preceding 1 year exacerbation history were used to categorise risk, whilst, either the highest COPD assessment test (CAT) or modified MRC Scale (mMRC) score was used to categorise symptoms. A change in FEV_1_ > 60 mls over 1 year was used to identify rapid decliners [7].

The following patient reported outcome measurements were performed; the CAT [8] and St George’s Respiratory Questionnaire (SGRQ-C) [9] for health status, the mMRC Scale for dyspnoea [10], and Centre for Epidemiological Studies Depression Scale (CES-D) for depression [11].

Lung function was assessed by spirometry, plethysmography and gas transfer and was performed in accordance with European Respiratory Society (ERS)/American Thoracic Society (ATS) recommendations [12–14]. Reversibility was performed using salbutamol 400mcg. Fat free mass (FFM) and fat free mass index (FFMI) were determined by bioelectrical impedance analysis. Functional capacity was assessed by the 6 min walk test (6MWT) and performed in accordance with ATS/ERS standard. [15] A practice walk was performed if patients had not performed a 6 min walk test over the preceding 1 year.

Spontaneous and/or induced sputum was processed for quantitative polymerase chain reaction (qPCR). Selected sputum plugs were homogenised with phosphate-buffered saline (PBS) with glass beads and qPCR was performed for the detection of the common respiratory potentially pathogenic microorganisms (PPM) *H. influenzae*, *M.catarrhalis* and *S. pneumoniae* as previously described [16]. The threshold for detection for pathogens by qPCR was 1 × 10^4^ copies per ml. Venepuncture was performed and the samples collected were sent to the local hospital laboratories for full blood counts (FBC) and C-reactive protein (CRP) analysis.

### Statistical analysis

The Kolmogorov–Smirnov test was applied to determine the normality of data. Differences in between groups were performed using unpaired t tests or Mann–Whitney test for parametric and non-parametric data respectively. Categorical variables were analysed using chi square test. *P* < 0.05 was considered statistically significant. Statistical analyses were performed using GraphPad Prism version 5.00 (San Diego, California; USA).

## Results

**Fig. 1:**
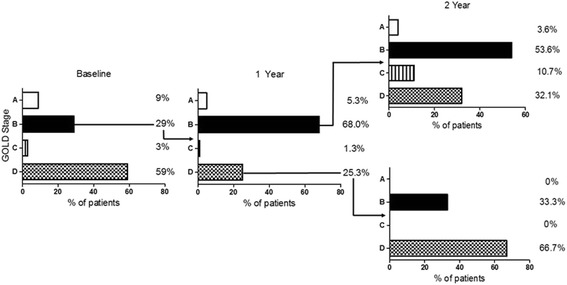
GOLD B progression across 2 years. 107 GOLD B patients at baseline; 75 remained at 1 year and 40 at 2 year. Of these 40 patients, 28 were categorised as stable and 12 unstable

Three hundred seventy patients completed the baseline visit; demographics are shown in Table 1. The GOLD categorisation using the higher of the two symptom scores was as follows; 9.2% in group A (*n* = 34), 28.9% in group B (*n* = 107), 3.2% in group C (*n* = 12) and 58.7% in group D (*n* = 217) (Fig. 1). Categorisation using only the CAT score showed that patients in groups A and C would remain in these groups, whilst 3.7% (4/107) of the group B and 2.3% (5/217) of the group D patients would be categorised into groups A and C respectively. Likewise if patients were categorised using only the mMRC score, all patients in group A and C remained in their groups, whilst 31.8% (34/107) of the group B and 24.9% (54/217) of the group D patients would be reclassified into groups A and C respectively.

**Table 1:** Baseline demographics of patients

Demographic	*n* = 370
Gender (% Male)	65
Age (years)	70.2 (8.8)
Smoking status (Current %)	30.0
Pack years	46.0 [10.0–220.0]
BMI (kg/m^2^)	26.2 [15.6–49.2]
Exacerbations (1 year prior)	1.0 [0.0–15.0]
Inhaled steroid use %	78.8
Oral steroid use %	3.8
LABA use %	70.7
LAMA use %	75.0
Azithromycin use %	3.5
Post BD FEV_1_ (L)	1.4 (0.6)
Post BD FEV_1_ %	57.0 [20.0–117.0]
Post BD Ratio	0.5 [0.2–0.8]
GOLD A n (%)	34 (9.2)
GOLD B n (%)	107 (28.9)
GOLD C n (%)	12 (3.2)
GOLD D n (%)	217 (58.7)

Summaries are presented as percentages, Mean (SD) or Median [Range] as appropriate

Definitions of abbreviations; *BMI* body mass index, *LABA* long acting beta agonist, *LAMA* long acting muscarinic antagonist, *Post BD* post bronchodilator, *FEV*_*1*_ forced expired volume in first second

Table 2 shows the baseline clinical characteristics of GOLD A and B patients. FEV_1_ % predicted was similar between the groups, despite the greater symptoms in GOLD B patients. There were more current smokers in group B (36.5% v 15.2% respectively, *p* = 0.031) and a significantly higher proportion of group B patients had chronic bronchitis (57.4% v 21.2%, *p* = 0.0003) and cardiovascular comorbidities (82.2% v 61.8% *p* = 0.019). The clinical characteristics of GOLD C and D patients are presented in the data supplement in Additional file 1: Table S1. The comparison of clinical characteristics between GOLD B and D patients are shown online in Additional file 2: Table S2; the number of exacerbations, symptoms burden and lung function were significantly worse in GOLD D patients compared to GOLD B.

**Table 2:** Baseline characteristics of patients categorised as GOLD A and B

	GOLD A (*n* = 34)	GOLD B (*n* = 107)	*p* value
Demographics			
Gender (% Male)	58.8	67.3	0.41
Pack years	44.5 [10.0–135.0]	45 [10.0–220.0]	0.53
Smoking status (% Current)	15.2	36.5	*0.031*
BMI (Kg/m^2^)	26.1 [20.6–47.0]	27.2 [16.5–49.2]	0.73
FFMI (Kg/m^2^)	17.3 [12.9–28.7]	17.6 [10.0–27.0]	0.92
Chronic bronchitis (%)	21.2	57.4	*0.0003*
Exacerbations (1 year prior)	0.0 [0.0–1.0]	0.0 [0.0–1.0]	0.11
Co-morbidities			
Cardiovascular comorbidity any (%)	61.8	82.2	*0.019*
Comorbidities any (%)	35.3	16.8	*0.03*
Patient reported outcomes			
SGRQ total	19.3 [1.0–40.0]	40.9 [5.7–82.4]	*<0.0001*
SGRQ symptoms	29.5 (16.5)	55.3 (17.8)	*<0.0001*
SGRQ impact	12.0 [3.0–24.0]	25.0 [0.0–75.5]	*<0.0001*
SGRQ activity	34.8 (18.5)	58.9 (22.6)	*<0.0001*
CAT	6.0 [2.0–9.0]	16.0 [4.0–39.0]	*<0.0001*
CES-D	5.0 [1.0–6.0]	11.5 [1.0–4.0]	*<0.0001*
Functional capacity			
6MWD (metres)	457.0 [169.0–636.0]	405.7 [119.0–702.0]	0.07
Lung function			
Vital capacity %	102.3 (22.5)	100.7 (17.9)	0.73
Total lung capacity %	108.9 (16.1)	109.2 (20.1)	0.96
Residual volume %	130.6 [89.1–179.9]	132.0 [63.0–286.0]	0.90
Inspiratory capacity %	87.1 (23.0)	89.5 (21.3)	0.65
FRC %	125.8 (24.0)	128.7 (33.2)	0.71
DLCO %	70.8 [56.0–116.5]	63.0 [28.0–106.0]	0.05
KCO %	86.8 [64.0–126.5]	76.4 [35.0–137.0]	0.13
VA %	84.2 (13.3)	80.8 (10.7)	0.25
Post FEV_1_ %	71.0 (15.2)	68.3 (11.4)	0.27
Reversibility %	9.8 [4.0–16.0]	6.7 [−3.0–+38.0]	0.13
Reversibility mls	150.0 [−40.0–+290.0]	100.0 [−80.0–+670.0]	0.21
Bacteriology			
Bacterial load (genome copies/ml)	7.11 × 10^6^ [0–1.81 × 10^10^]	7.74 × 10^4^ [0–4.91 × 10^8^]	0.29
Colonised (% >1 × 10^4^ total PPM)	63.7	55.6	0.75
Systemic inflammation			
CRP (mg/L)	3.0 [1.0–22.0]	3.0 [1.0–157.0]	0.35
WBC (10^9^/L)	6.8 [3.6–12.0]	7.3 [5.0–12.6]	0.11
Eosinophils (10^9^/L)	0.2 [0.0–0.8]	0.2 [0.0–0.9]	0.83

Summaries are presented as mean (SD), percentage or Median [Range] as appropriate

Definitions of abbreviations: *BMI* body mass index, *FFMI* fat free mass index, *SGRQ* St George’s Respiratory Questionnaire, *CAT* COPD Assessment Test, *CES-D* Centre for Epidemiologic Studies Depression, *6MWD* six minute walk distance, *FRC* functional residual capacity, *DLCO* diffusing capacity of the lungs for carbon monoxide, *KCO* carbon monoxide transfer coefficient, *VA* alveolar volume, *CRP* C-reactive protein, *WBC* white blood count, *PPM* potentially pathogenic microorganism

Seventy five out of 107 GOLD B patients attended for 1 year follow up; the major reasons for this decrease in numbers were loss to follow up and withdrawal of consent. The majority of patients were stable and remained in group B at 1 year (68.0%). There were 19 unstable GOLD B patients (25.3%) who progressed to GOLD D; 8 patients due to a decline in FEV_1,_ 10 patients due to an increase in exacerbation risk, and 1 subject displayed both of these characteristics.

**Fig. 2:**
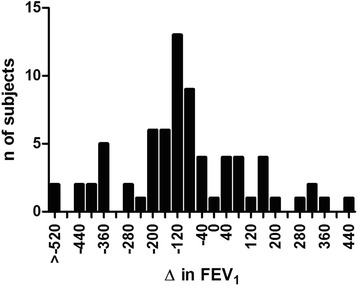
Change in post bronchodilator FEV_1_ in GOLD B over 1 year. 58.7% of GOLD B patients displayed rapid decline in FEV_1_

The mean change in FEV_1_ over 1 year was a decline of 66mls (Fig. 2). There was no decline in FEV_1_ in 23 patients, whilst 44 patients (58.7%) displayed a decline greater than 60mls over the year.

At 2 year follow up, 40 out of 107 GOLD B patients remained in the study. Of those patients who remained stable at 1 year (*n* = 28), the majority remained in group B (*n* = 15; 53.6%), with a third progressing to group to D (*n* = 9; 32.1%). Of the 19 patients who deteriorated to GOLD D at 1 year, 12 remained in the study at 2 years. The majority of patients (*n* = 8) remained in group D, while the remaining patients (*n* = 4) reverted to group B (Fig. 1).

Table 3 shows the clinical characteristics at 1 year of patients who remained in group B at 1 year and those who progressed to group D (stable and unstable patients respectively). At 1 year, unstable patients had significantly more exacerbations over the previous year (means 1.5 v 0.2, *p* = <0.0001), higher mean CAT scores (20.0 v 15.5, *p* = 0.018), worse mean SGRQ total scores (50.0 v 41.1, *p* = 0.001), higher mean C-reactive protein levels (5.0 v 3.0, *p* = 0.017), and reduced mean 6MWD (347.3 v 440.5, *p* = 0.023).

**Table 3:** Comparison of 1 year characteristics of patients who remained stable (GOLD B) and unstable patients (GOLD D) from baseline

	GOLD B (*n* = 51)	GOLD D (*n* = 19)	*p* - value
Demographics			
Gender (% Male)	75.6	52.6	0.15
BMI (Kg/m^2^)	27.0 [16.2–44.8]	27.7 [21.7–48.5]	0.32
FFMI (Kg/m^2^)	18.3 [8.8–24.0]	17.6 [11.7–27.0]	0.75
Exacerbations (1 year prior)	0.0 [0.0–1.0]	2.0 [0.0–4.0]	*<0.0001*
Patient reported outcomes			
SGRQ total	41.1 (15.5)	50.0 [22.2–82.3]	*0.001*
SGRQ symptoms	54.6 (19.9)	61.0 (17.5)	0.24
SGRQ impact	26.3 [2.1–71.6]	42.4 [4.6–60.1]	0.05
SGRQ activity	59.3 [0.0–100.0]	78.1 [22.5–100.0]	*0.003*
CAT	15.5 [9.0–35.0]	20.0 [9.0–37.0]	*0.018*
CES-D	12.0 [0.0–33.0]	15.0 [0.0–31.0]	0.58
Functional capacity			
6MWD (metres)	440.5 (125.7)	347.3 (123.4)	*0.023*
Lung function			
Vital capacity %	99.2 (21.9)	93.6 (13.1)	0.40
Total lung capacity %	110.2 (17.0)	110.5 (23.5)	0.96
Residual volume %	132.0 (30.4)	150.0 (55.9)	0.16
Inspiratory capacity %	93.1 (21.8)	91.4 (20.9)	0.81
FRC %	125.6 (25.7)	132.5 (45.9)	0.52
DLCO %	56.5 [40.0–102.0]	61.5 [32.0–115.0]	0.87
KCO %	75.5 (80.1)	74.7 (85.0)	0.53
VA %	81.4 (13.1)	76.9 (14.2)	0.29
Post FEV_1_ %	68.2 (12.3)	57.3 (12.8)	*0.002*
Reversibility %	−4.4 [−53.8–+8.0]	−8.1 [17.3–+8.3]	0.25
Reversibility mls	20.0 [−170.0–580]	90 [−120–200]	0.90
Change in FEV_1_ from baseline	−80.0 [−420.0–+939.0]	−120.0 [−531.0–+180.0]	0.07
Bacteriology			
Bacterial load (genome copies/ml)	2.36 × 10^6^ [0–8.89 × 10^8^]	10.3 × 10^7^ [0–1.76 × 10^8^]	0.25
Colonised (% >1 × 10^4^ total PPM)	71.4	77.2	1.00
Systemic inflammation			
CRP (mg/L)	3.0 [1.0–20.0]	5.0 [2.0–20.0]	*0.017*
WBC (10^9^/L)	6.9 [3.6–13.5]	7.5 [4.8–11.8]	0.73
Eosinophils (10^9^/L)	0.2 [0.1–0.6]	0.2 [0.0–0.4]	0.24

Summaries are presented as mean (SD), percentage or Median [Range] as appropriate

Definitions of abbreviations: *BMI* body mass index, *FFMI* fat free mass index, *SGRQ* St George’s Respiratory Questionnaire, *CAT* COPD assessment test, *CES-D* Centre for Epidemiologic Studies Depression, *6MWD* six minute walk distance, *FRC* functional residual capacity, *DLCO* diffusing capacity of the lungs for carbon monoxide, *KCO* carbon monoxide transfer coefficient, *VA* alveolar volume, *CRP* C-reactive protein, *WBC* white blood count, *PPM* potentially pathogenic microorganisms

Table 4 shows the characteristics of the stable and unstable patients at the baseline visit. Unstable patients had a significantly lower mean FEV_1_ % predicted (69.7% v 62.4%; *p* = 0.016), worse mean SGRQ score (40.1 v 50.0; *p =* 0.019) and mean CAT score (14.0 v 21.0; *p* = 0.006), and higher mean CRP levels with a trend towards significance (3.0 v 4.5; *p* = 0.06). Patients who declined due to FEV_1_ change had lower FEV_1_ at baseline (means 55.7% v 67.8%; *p* = 0.0023), and a much greater magnitude of FEV_1_ deterioration over 1 year (284 mls v 44 mls; *p* = 0.0021), and were more likely to be current smokers (62.5% v 10.0%; *p* = 0.04) (see Additional file 3: Table S3 in the data supplement).

**Table 4:** Baseline characteristics of patients who remained stable (GOLD B) and unstable patients (GOLD D) at 1 year

	Stable (*n* = 51)	Unstable (*n* = 19)	*p* - value
Demographics			
Gender (% Male)	72.5	52.6	0.15
Pack years	45.0 [10.0–220.0]	52.0 [10.0–113.0]	0.67
Smoking status (% Current)	37.3	36.8	1.00
BMI (Kg/m^2^)	27.2 [17.3–49.2]	27.9 [20.7–45.0]	0.28
FFMI (Kg/m^2^)	17.8 (3.4)	18.7 (3.4)	0.34
Chronic bronchitis (%)	58.3	61.1	1.00
Exacerbations (1 year prior)	0.0 [0.0–1.0]	0.0 [0.0–1.0]	0.88
Co-morbidities			
Cardiovascular comorbidity any (%)	94.1	94.7	1.00
Comorbidities any (%)	94.1	94.7	1.00
Patient reported outcomes			
SGRQ total	40.1 [14.6–80.0]	50.0 [22.2–82.3]	*0.019*
SGRQ symptoms	55.1 [22.0–91.0]	60.8 [15.4–90.0]	0.10
SGRQ impact	26.0 [2.5–71.6]	35.9 [9.0–73.7]	0.20
SGRQ activity	59.3 [7.6–100.0]	79.0 [15.6–100.0]	*0.003*
CAT	14.0 [4.0–28.0]	21.0 [8.0–39.0]	*0.006*
CES-D	11.0 [1.0–32.0]	12.5 [3.0–38.0]	0.94
Functional capacity			
6MWD (metres)	425.3 (98.5)	361.5 (124.8)	0.06
Lung function			
Vital capacity %	103.3 (19.8)	96.6 (16.5)	0.25
Total lung capacity %	108.8 (19.9)	111.8 (24.6)	0.65
Residual volume %	130.9 (37.4)	150.4 (60.2)	0.16
Inspiratory capacity %	87.0 (23.0)	89.8 (16.1)	0.67
FRC %	129.3 (30.7)	134.2 (44.5)	0.65
DLCO %	66.2 (20.3)	64.1 (24.3)	0.76
KCO %	75.5 [41.2–137.0]	81.5 [35.0–124.0]	0.97
VA %	82.8 (12.0)	79.2 (9.3)	0.32
Post FEV_1_ %	69.7 (11.7)	62.4 (9.0)	*0.016*
Reversibility %	5.0 [−3.0–+33.0]	7.4 [0.8–32.0]	0.47
Reversibility mls	90.0 [−40.0–+670.0]	95.0 [20.0–+380.0]	0.90
Bacteriology			
Bacterial load (genome copies/ml)	1.44 × 10^5^ [0.0–2.43 × 10^8^]	7.4 × 10^2^ [0–10.4 × 10^7^]	0.66
Colonised (% >1 × 10^4^ total PPM)	66.7	46.2	0.30
Systemic inflammation			
CRP (mg/L)	3.0 [1.0–35.5]	4.5 [1.0–157.0]	0.06
WBC (10^9^/L)	7.2 [5.4–12.6]	7.4 [5.0–12.6]	0.42
Eosinophils (10^9^/L)	0.2 [0.0–0.9]	0.2 [0.1–0.3]	0.32

Summaries are presented as mean (SD), percentage or Median [Range] as appropriate

Definitions of abbreviations: *BMI* body mass index, *FFMI* fat free mass index, *SGRQ* St George’s Respiratory Questionnaire, *CAT* COPD Assessment Test, *CES-D* Centre for Epidemiologic Studies Depression, *6MWD* six minute walk distance, *FRC* functional residual capacity, *DLCO* diffusing capacity of the lungs for carbon monoxide, *KCO* carbon monoxide transfer coefficient, *VA* alveolar volume, *CRP* C-reactive protein, *WBC* white blood count, *PPM* potentially pathogenic microorganisms

## Discussion

The criteria used to define GOLD B patients results in a degree of homogeneity in terms of symptoms, lung function and exacerbation history when assessed at a single visit. However, longitudinal follow up reveals a heterogeneous course of disease. GOLD B patients who progressed to GOLD D had more severe disease characteristics at the start of the 1 year follow up. This unstable subgroup had significantly worse CAT and SGRQ scores and lower FEV_1_ compared to stable patients at baseline. Progression of GOLD B to GOLD D appears to be more likely in highly symptomatic patients.

Approximately equal numbers of unstable patients deteriorated due to FEV_1_ alone or exacerbations alone (8 versus 10 respectively). Although these are small groups to perform sub-analysis, there were very clear differences in the rate of FEV_1_ decline between these groups (means 284 versus 44 mls respectively), and there were more current smokers in the former group. The role of current smoking in promoting lung function decline is known [4], and we show here a subgroup of group B patients where current smoking is associated with a very rapid rate of FEV_1_ decline. The progression to group D in these patients was associated with both a lower FEV_1_ at baseline and a large decrease in FEV_1_ over 1 year (mean 284 mls). This indicates that switching from group B to D because of FEV_1_ criteria is not simply due to small changes in lung function that might occur in patients with lung function measurements just above the FEV_1_ 50% predicted threshold.

The worse patient reported outcome scores at baseline in unstable group B patients suggest increased disease activity. The deterioration of group B patients towards group D is likely to have started before the baseline visit in many of these patients. The high symptom burden is probably related to increased disease activity associated with rapid FEV_1_ decline. Alternatively, for patients who progressed to group D because of exacerbations, the higher symptom burden may be due to mild or unreported exacerbations in the previous year, which subsequently evolved to moderate to severe exacerbations during the follow up period.

Different thresholds have been used to define COPD patients with a rapid decline in FEV_1_; 40 mls/year has been suggested [17], while 60 mls/year has also been used [7]. We observed a mean decrease of 66 mls/year, with 58.7% of patients showing >60 ml decline. This is a higher mean rate of FEV_1_ decline than previously observed in many cohort studies and clinical trials [18, 19]. However, Koskela et al. [20] recently reported one third of patients with rapid declining FEV_1_ had a mean decrease of 78 mls/year compared to the 28 mls/year in the rest of the patients. Here we focused just on group B patients. FEV_1_ decline is known to vary with GOLD stage 1–4 and with GOLD group A-D. Goosens et al. [18] reported a mean 48 mls/year decline in FEV_1_ in GOLD B patients, while Kim et al. [19] reported a smaller mean decline of 27 mls/year in GOLD B patients. Our higher rate of decline compared to these previous reports in group B patients may reflect the recruitment strategy used, focusing on hospital clinics and therefore recruiting more severe/symptomatic patients. This lung function decline could not be explained by changes in medication as patients remained on their normal medications when entering this observational study.

In the ECLIPSE study, 36% of group B patients remained stable, while 35% progressed to group D after 1 year and 22% improved to group A [2]. In our COPDMAP cohort, a slightly lower proportion (25%) progressed to group D, but many more remained stable (68%). ECLIPSE was a worldwide study while COPDMAP is a UK study, and these differences may simply reflect the varied healthcare of the patients in different studies and locations.

Significantly more group B patients were current smokers compared to group A. Furthermore, group B had more comorbidities and a greater prevalence of chronic bronchitis compared to group A patients. Despite these differences, and a higher symptom burden in group B, there were no differences in lung function between groups. Similarly, previous studies have reported similar lung function in group B compared to group A patients [19]. These findings highlight that the increased symptom burden, including increased breathlessness, in group B patients is not attributed to airflow obstruction alone, and further underscores the poor relationship between lung function and symptoms in COPD patients. The increased symptom burden in GOLD B patients is likely to be due, at least in part, to the increased prevalence of co-morbidities.

Other cohort studies have not reported lower rates of current smoking in group A patients compared to group B [19, 21, 22]. Our relatively small number of group A patients means that the estimate of current smokers in our study is not robust.

We only had a small number of patients who attended for 2 year follow up, but nevertheless the pattern of approximately 25% further switching to group D in the second year was observed. Furthermore, the majority of patients who had progressed to group D after 1 year remained in this category at 2 years.

A key strength of the current study is the detailed characterisation of the GOLD B group. Potential limitations are the sample sizes for subgroup analysis, and the dropout rate during longitudinal follow up.

## Conclusion

In conclusion, we show that a subset of GOLD B patients who moved to the higher risk D category after 1 year had greater symptoms and worse health status at the start of the study. Patient reported outcome scores appear to be related to the risk of disease progression in this GOLD category. Within GOLD B there also appears to be a subgroup with a very high rate of lung function decline associated with current smoking. These findings demonstrate the heterogeneous nature of GOLD B patients during longitudinal follow up, and that this group of patients who are at risk of disease progression should be carefully followed-up in clinical practice.

## Additional files

Additional file 1: Table S1. Baseline characteristics of patients categorised as GOLD C and D. (DOCX 17 kb)
Additional file 2: Table S2. Comparison of baseline demographics, symptoms and lung function between patients categorised as GOLD B and D. (DOCX 17 kb)
Additional file 3: Table S3. Baseline characteristics of patients who declined to GOLD D, according to reason (FEV_1_ and Exacerbation). (DOCX 18 kb)

## Abbreviations

6MWT: 6 minute walk test
ATS: American Thoracic Society
BMI: Body mass index
CAT: COPD assessment test
CES-D: Centre for Epidemiological Studies Depression Scale
COPD: Chronic obstructive pulmonary disease
CRP: C-reactive protein
ERS: European Respiratory Society
FBC: Full blood counts
FEV_1_: Forced expiratory volume in 1 second
FFM(I): Fat free mass (index)
FVC: Forced vital capacity
GOLD: Global Initiative for Chronic Obstructive Pulmonary Disease
mMRC: Modified MRC scale
PBS: Phosphate buffered saline
PPM: Potentially pathogenic microorganisms
qPCR: Quantitative polymerase chain reaction
SGRQ-C: St George’s respiratory questionnaire
