# Supplementary material for: Characteristics and longitudinal progression of chronic obstructive pulmonary disease in GOLD B patients
Source: BMC Pulm Med. 2017 Feb 20;17:42. doi: 10.1186/s12890-017-0384-8 (PMC5319137; doi:10.1186/s12890-017-0384-8)
Supplement: Additional file 1: Table S1. — Baseline characteristics of patients categorised as GOLD C and D. (DOCX 17 kb) [file 12890_2017_384_MOESM1_ESM.docx]

**Table S1. Baseline characteristics of patients categorised as GOLD C and D**

|  | GOLD C (n=12) | GOLD D (n=217) |
| --- | --- | --- |
| ***Demographics*** |  |  |
| Gender (% Male) | 58.3 | 65.9 |
| Pack Years | 40 [22.0 – 74.0] | 47.0 [10.0 – 201.0] |
| Smoking Status (% Current) | 8.3 | 29.0 |
| BMI (Kg/m^2^) | 23.6 [15.6 – 35.4] | 26.1 [17.5 – 42.7] |
| FFMI (Kg/m^2^) | 17.1 [8.9 – 19.3] | 16.7 [6.6 – 29.9] |
| Chronic Bronchitis (%) | 16.7 | 61.8 |
| Exacerbations (1 year prior) | 2.0 [1.0 – 4.0] | 2.0 [0.0 -15.0] |
| ***Co-morbidities*** |  |  |
| Cardiovascular Comorbidity Any (%) | 83.3 | 72.3 |
| Comorbidities Any (%) | 25.0 | 25.8 |
| ***Patient Reported Outcomes*** |  |  |
| SGRQ Total | 32.1 [14.4 – 46.0] | 54.0 [15.0 – 87.9] |
| SGRQ Symptoms | 46.2 (13.6) | 66.1 (17.8) |
| SGRQ Impact | 19.3 [1.0 – 42.0] | 36.0 [3.0-18.1] |
| SGRQ Activity | 46.4 (14.7) | 72.3 (20.2) |
| CAT | 5.0 [2.0 -9.0] | 20.0 [2.0 – 37.0] |
| CES-D | 8.0 [2.0 - 14.0] | 12.0 [1.0 – 47.0] |
| ***Functional Capacity*** |  |  |
| 6MWD (metres) | 446.8 (152.6) | 366.9 (107.6) |
| ***Lung Function*** |  |  |
| Vital Capacity % | 93.2 (15.1) | 91.7 (21.1) |
| Total Lung Capacity % | 105.7 (23.9) | 116.7 (18.3) |
| Residual Volume % | 120.0 [87.0 – 213.1] | 165.0 [64.0 – 303.0] |
| Inspiratory Capacity % | 74.2 (10.1) | 81.1 (26.1) |
| FRC % | 134.7 (52.8) | 150.9 (34.5) |
| DLCO % | 72.0 [50.0 – 80.0] | 49.0 [20.0 – 112.0] |
| KCO % | 77.0 [57.0 -113.0] | 67.0 [27.0 – 132.0] |
| VA % | 82.5 (12.1) | 77.8 (14.3) |
| Post FEV_1_ % | 56.1 (12.3) | 48.3 (17.8) |
| Reversibility % | N/A | 9.8 [-13.0 – +51.0] |
| Reversibility mls | N/A | 100 [-180.0 – +420.0] |
| ***Systemic Inflammation*** |  |  |
| CRP (mg/L) | 1.5 [1.0 – 13.0] | 3.0 [1.0 – 70.0] |
| WBC (10^9^/L) | 7.2 [4.5 – 10.2] | 7.4 [4.0 – 15.6] |
| Eosinophils (10^9^/L) | 0.1 [0.0 – 0.3] | 0.2 [0.0 – 1.5] |

*Summaries are presented as mean (SD), percentage or Median [Range] as appropriate. N/A as data were only available for n= 3 subjects.*

*Definitions of abbreviations: BMI = body mass index; FFMI = fat free mass index; SGRQ = St George’s Respiratory Questionnaire; CAT = COPD Assessment Test; CES-D = Centre for Epidemiologic Studies Depression; 6MWD = Six Minute Walk Distance; FRC = Functional Residual Capacity; DLCO = Diffusing capacity of the lungs for carbon monoxide; KCO = Carbon monoxide transfer coefficient; VA = Alveolar Volume CRP = C-reactive Protein; WBC = White blood count*
