# Supplementary material for: Characteristics and longitudinal progression of chronic obstructive pulmonary disease in GOLD B patients
Source: BMC Pulm Med. 2017 Feb 20;17:42. doi: 10.1186/s12890-017-0384-8 (PMC5319137; doi:10.1186/s12890-017-0384-8)
Supplement: Additional file 2: Table S2. — Comparison of baseline demographics, symptoms and lung function between patients categorised as GOLD B and D. (DOCX 17 kb) [file 12890_2017_384_MOESM2_ESM.docx]

**Table S2. Comparison of baseline demographics, symptoms and lung function between patients categorised as GOLD B and D**

|  | GOLD B (n=107) | GOLD D (n=217) | p value |
| --- | --- | --- | --- |
| ***Demographics*** |  |  |  |
| Gender (% Male) | 67.3 | 65.9 | 0.90 |
| Pack Years | 45 [10.0 - 220.0] | 47 [10 - 201] | 0.61 |
| Smoking Status (% Current) | 36.5 | 29.0 | 0.20 |
| BMI (Kg/m^2^) | 27.2 [16.5 - 49.2] | 26.1 [17.5 – 42.7] | 0.08 |
| FFMI (Kg/m^2^) | 17.6 [10.0 - 27.0] | 16.7 [6.6 – 29.9] | *0.041** |
| Chronic Bronchitis (%) | 57.4 | 61.8 | 0.32 |
| Exacerbations (1 year prior) | 0.0 [0.0 -1.0] | 2.0 [0.0 – 15.0] | *<0.0001** |
| ***Patient Reported Outcomes*** |  |  |  |
| SGRQ Total | 40.9 [5.7 - 82.4] | 54.0 [15.0 – 87.9] | *<0.0001** |
| SGRQ Symptoms | 55.3 (17.8) | 66.1 (17.8) | *<0.0001** |
| SGRQ Impact | 25.0 [0.0 - 75.5] | 36.0 [3.0-18.1] | *<0.0001** |
| SGRQ Activity | 58.9 (22.6) | 72.3 (20.2) | *<0.0001** |
| CAT | 16.0 [4.0 - 39.0] | 20.0 [2.0 – 37.0] | *0.003**  33 |
| CES-D | 11.5 [1.0 - 4.0] | 12.0 [1.0 – 47.0] | *<0.0001** |
| ***Lung Function*** |  |  |  |
| Vital Capacity % | 100.7 (17.9) | 91.7 (21.1) | *0.002** |
| Total Lung Capacity % | 109.2 (20.1) | 116.7 (18.3) | *0.005** |
| Residual Volume % | 132.0 [63.0 - 286.0] | 165.0 [64.0 – 303.0] | *<0.0001** |
| Inspiratory Capacity % | 89.5 (21.3) | 81.1 (26.1) | *0.002** |
| FRC % | 128.7 (33.2) | 150.9 (34.5) | *<0.0001** |
| DLCO % | 63.0 [28.0 - 106.0] | 49.0 [20.0 – 112.0] | *<0.0001** |
| KCO % | 76.4 [35.0 - 137.0] | 67.0 [27.0 – 132.0] | *0.0006** |
| VA % | 80.8 (10.7) | 77.8 (14.3) | 0.12 |
| Post FEV_1_ % | 68.3 (11.4) | 48.3 (17.8) | *<0.0001** |
| Reversibility % | 6.7 [-3.0 - +38.0] | 9.8 [-13.0 – 51.0] | 0.23 |
| Reversibility mls | 100.0 [-80.0 - +670.0] | 100 [-180.0 – 420.0] | 0.46 |

*Summaries are presented as mean (SD), percentage or Median [Range] as appropriate.*

*Definitions of abbreviations: BMI = body mass index; FFMI = fat free mass index; SGRQ = St George’s Respiratory Questionnaire; CAT = COPD Assessment Test; CES-D = Centre for Epidemiologic Studies Depression; FRC = Functional Residual Capacity; DLCO = Diffusing capacity of the lungs for carbon monoxide; KCO = Carbon monoxide transfer coefficient; VA = Alveolar Volume*
