# Supplementary material for: Characteristics and longitudinal progression of chronic obstructive pulmonary disease in GOLD B patients
Source: BMC Pulm Med. 2017 Feb 20;17:42. doi: 10.1186/s12890-017-0384-8 (PMC5319137; doi:10.1186/s12890-017-0384-8)
Supplement: Additional file 3: Table S3. — Baseline characteristics of patients who declined to GOLD D, according to reason (FEV1 and Exacerbation). (DOCX 18 kb) [file 12890_2017_384_MOESM3_ESM.docx]

**Table S3. Baseline characteristics of patients who declined to GOLD D, according to reason (FEV_1_ and Exacerbation)**

|  | FEV_1_ (n = 8) | Exac (n = 10) | p – value |
| --- | --- | --- | --- |
| ***Demographics*** |  |  |  |
| Gender (% Male) | 62.5 | 50.0 | 0.66 |
| Pack Years | 51.3 (32.7) | 58.7 (33.8) | 0.64 |
| Smoking Status (% Current) | 62.5 | 10.0 | *0.043** |
| BMI (Kg/m^2^) | 28.3 (3.3) | 30.4 (8.2) | 0.52 |
| FFMI (Kg/m^2^) | 18.8 (2.6) | 19.0 (4.3) | 0.93 |
| Chronic Bronchitis (%) | 71.4 | 50.0 | 0.62 |
| Exacerbations (1 year prior) | 0 [0-1] | 0.5 [0-1] | 0.66 |
| ***Co-morbidities*** |  |  |  |
| Cardiovascular Comorbidity Any (%) | 100.0 | 90.0 | 1.00 |
| Comorbidities Any (%) | 100.0 | 90.0 | 1.00 |
| ***Patient Reported Outcomes*** |  |  |  |
| SGRQ Total | 47.6 (14.4) | 57.6 (17.2) | 0.39 |
| SGRQ Symptoms | 60.5 (7.4) | 60.8 (23.2) | 0.98 |
| SGRQ Impact | 41.0 [11-46] | 40.0 [4.6-60.1] | 0.41 |
| SGRQ Activity | 66.0 [53-100.0] | 88.0 [22.5-100.0] | 0.31 |
| CAT | 20.0 (8.4) | 21.2 (5.8) | 0.72 |
| mMRC | 3.0 [1.0-3.0] | 3.0 [1.0-4.0] | 0.87 |
| CES-D | 14.0 (8.9) | 13.9 (10.3) | 0.98 |
| ***Functional Capacity*** |  |  |  |
| QMVC (kg) | 30.3 [12.0-40.0] | 24.7 [13.3-69.4] | 0.90 |
| 6MWD (metres) | 396.0 [210.0-510.0] | 332.0 [119.0-563.0] | 0.37 |
| ***Lung Function*** |  |  |  |
| Vital Capacity % | 92.3 (9.9) | 97.7 (19.7) | 0.58 |
| Total Lung Capacity % | 116.6 (17.0) | 106.8 (27.6) | 0.48 |
| Residual Volume % | 148.1 [131-234.7] | 119.0 [69.0-286.0] | 0.16 |
| Inspiratory Capacity % | 82.9 (9.3) | 93.7 (18.7) | 0.25 |
| FRC % | 147.2 (38.2) | 121.6 (44.4) | 0.29 |
| DLCO % | 56.7 (18.9) | 67.7 (26.8) | 0.53 |
| KCO % | 72.6 (27.6) | 86.8 (31.0) | 0.49 |
| Post FEV_1_ % | 55.7 (3.4) | 67.8 (8.9) | *0.0023** |
| ***Bacteriology*** |  |  |  |
| Bacterial Load (genome copies/ml) | 0.0 [0.0 - 1.04x10^8^] | 1.05x10^6^ [0 - 5.52x10^7^] | 0.87 |
| Colonised (% >1x10^4^ total PPM) | 40.0 | 42.9 | 1.00 |
| ***Systemic Inflammation*** |  |  |  |
| CRP (mg/L) | 4.0 [1.0-15.0] | 5.5 [3.0-157.0] | 0.35 |
| WBC (10^9^/L) | 8.3 (2.7) | 7.7 (2.3) | 0.62 |
| Eosinophils (10^9^/L) | 0.2 (0.1) | 0.2 (0.1) | 0.17 |

*Summaries are presented as mean (SD), percentage or Median [Range] as appropriate.*

*Definitions of abbreviations: BMI = body mass index; FFMI = fat free mass index; SGRQ = St George’s Respiratory Questionnaire; CAT = COPD Assessment Test; CES-D = Centre for Epidemiologic Studies Depression; 6MWD = Six Minute Walk Distance; FRC = Functional Residual Capacity; DLCO = Diffusing capacity of the lungs for carbon monoxide; KCO = Carbon monoxide transfer coefficient; CRP = C-reactive Protein; WBC = White blood count; PPM = potentially pathogenic microorganisms*
